# Weaning Failure in Critically Ill Patients Is Related to the Persistence of Sepsis Inflammation

**DOI:** 10.3390/diagnostics12010092

**Published:** 2021-12-31

**Authors:** Anna Kyriakoudi, Nikoletta Rovina, Ourania Koltsida, Eirini Kostakou, Elissavet Konstantelou, Matina Kardara, Maria Kompoti, Anastasios Palamidas, Georgios Kaltsakas, Antonia Koutsoukou

**Affiliations:** 1 1st Department of Respiratory Medicine, Medical School, National and Kapodistrian University of Athens and “Sotiria” Chest Disease Hospital, 11527 Athens, Greece; annkyr@gmail.com (A.K.); rankoltsida@gmail.com (O.K.); ekostakou@yahoo.com (E.K.); eliskonst@yahoo.gr (E.K.); palamidastasos@gmail.com (A.P.); Georgios.Kaltsakas@gstt.nhs.uk (G.K.); koutsoukou@yahoo.gr (A.K.); 2 1st Department of Critical Care Medicine & Pulmonary Services, National and Kapodistrian University of Athens, Medical School, Evangelismos Hospital, 10676 Athens, Greece; kardara.matina@gmail.com; 3 Intensive Care Unit, General Hospital of Eleusis Thriasio, 13674 Athens, Greece; mariakompoti@gmail.com

**Keywords:** sepsis, inflammation, IL-18, IL-18BP, critically ill patients

## Abstract

Introduction: Septic patients undergoing mechanical ventilation (MV) often experience difficulty in weaning. Th aim of this study was to determine whether inflammatory biomarkers of sepsis could be indicative of the failure or success of spontaneous breathing trial (SBT) in these patients. Methods: Sixty-five patients on MV (42 septic and 23 intubated for other reasons) fulfilling the criteria for SBT were included in the study. Blood samples were collected right before, at the end of (30 min) and 24 h after the SBT. Serum inflammatory mediators associated with sepsis (IL-18, IL-18BP, TNF) were determined and correlated with the outcome of SBT. Results: A successful SBT was achieved in 45 patients (69.2%). Septic patients had a higher percentage of SBT failure as compared to non-septic patients (85% vs. 15%, *p* = 0.026), with an odds ratio for failing 4.5 times (OR = 4.5 95%CI: 1.16–17.68, *p* 0.022). IL-18 levels and the relative mRNA expression in serum were significantly higher in septic as compared to non-septic patients (*p* < 0.05). Sepsis was independently associated with higher serum IL-18 and TNF levels in two time-point GEE models (53–723, *p* = 0.023 and 0.3–64, *p* = 0.048, respectively). IL-18BP displayed independent negative association with rapid shallow breathing index (RSBI) (95% CI: −17.6 to −4, *p* = 0.002). Conclusion: Sustained increased levels of IL-18 and IL-18BP, acknowledged markers of sepsis, were found to be indicative of SBT failure in patients recovering from sepsis. Our results show that, although subclinical, remaining septic inflammation that sustaines for a long time complicates the weaning procedure. Biomarkers for the estimation of the septic burden and the right time for weaning are needed.

## 1. Introduction

Sepsis is a major cause of morbidity and mortality in critically ill patients [1]. Septic patients are dependent on mechanical ventilation (MV) for longer periods, and usually experience more weaning difficulties than patients without sepsis [2]. It is reported that critically ill patients with sepsis are 2.4 times more likely to fail spontaneous breathing trial (SBT) and require more weaning days (3.8 days) compared to critically ill patients without sepsis (2.5 days) [2], facts that seem to be associated with the rapid and shallow breathing of these patients on SBT while recovering from sepsis and respiratory failure [2,3,4,5,6].

Several markers of inflammation, such as interleukin-6 (IL-6), IL-18 and C-reactive protein (CRP), have been correlated with poor prognosis in septic patients [7,8,9,10,11,12,13,14,15,16]. Specifically, IL-18, a pleiotropic pro-inflammatory cytokine with abnormal expression in sepsis and a marker of inflammasome activity, has a documented value as a prognostic indicator for the outcome of critically ill septic patients [17,18,19,20]. IL-18, a cytokine upstream of the inflammatory cascade reaction, induces the massive synthesis of inflammatory cytokines (TNF, IL-1β) [21], which are the key mediators of sepsis-induced organ injury. These pro-inflammatory cytokines initiate the inflammatory responses involved in the progression of sepsis. Furthermore, IL-18 may also activate neutrophils to induce ROS generation [22], thereby damaging the vascular endothelium and basal membrane, eventually accelerating organ damage. IL-18 has been shown to be an effective indicator for monitoring treatment efficacy and patient recovery in patients with sepsis [23]. Rogers et al. [19], as well as Mierzchala-Pasierb et al. [20], have shown that increased IL-18 levels in critically ill patients correlated with disease severity and predicted ICU mortality. Furthermore, besides the septic parameter, Wu et al. showed that NLRP3 inflammasome may sense lung alveolar stretch to induce the release of downstream pro-inflammatory cytokines, contributing to the mechanism of lung inflammatory injury during mechanical ventilation [24].

IL-18BP, a constitutively secreted protein, has an exceptionally high affinity for IL-18 [25,26]. It is suggested that IL-18BP, by binding to IL-18, plays a central role in blunting Th1 response to organisms to down-regulate the triggering of responses to routine infections [27]. As the concentration of IL-18BP increases and binds IL-18, IL-18 possibly becomes less available as a pro-inflammatory cytokine.

Timely weaning from MV remains an important goal in critically ill patients. However, there are limited data on the potential role of biomarkers for the estimation of the right time to wean successfully from MV, especially in septic patients. Therefore, based on the central role of IL-18 as a prognostic marker of disease severity and outcome in septic patients, the aim of this study was to assess whether IL-18 and IL-18BP serum levels throughout spontaneous breathing trial (SBT) could possibly serve as biomarkers of weaning outcome in septic patients.

## 2. Materials and Methods

The study was performed in the ICU of the First Pulmonary and Critical Care Department, National and Kapodistrian University of Athens, in “Sotiria” Chest hospital, between January and December of 2018. Sixty-five patients (42 septic and 23 non-septic patients intubated for other reasons) mechanically ventilated ≥48 h that fulfilled the criteria for SBT were included after obtaining a signed informed consent from their representatives. Eighteen patients had diabetes mellitus as co-morbidity, 21 had hypertension, 9 had coronary disease, 5 had atrial fibrillation, 8 had COPD, 5 had chronic renal failure and 10 had lung cancer.

According to the usual practice of our institution the weaning process consisted of SBT of 30 min duration. The inclusion criteria for the study met the criteria for the initiation of SBT:

○ Improvement or resolution of causes leading to acute respiratory failure;
○ Partial Arterial Oxygen tension (PO_2_) > 60 mmHg with inspired Oxygen fraction (FiO_2_) < 0.4 and Peep 8 cm H_2_O;
○ Normal consciousness after discontinuation of sedation (CGS > 9);
○ Without fever (temperature > 38 °C) or hypothermia (temperature < 35 °C);
○ Hgb > 8md/gL;
○ Without hemodynamic instability or need for vasopressors.

Exclusion criteria were:

○ Intubation and mechanical ventilation for less than 48 h;
○ Age under 18 years old;
○ Tracheostomy (because of need for prolonged weaning);
○ Collaborative patients;
○ Patients with active upper gastrointestinal bleeding;
○ Immunocompromised patients or with human immunodeficiency virus (HIV);
○ Patients receiving a sedative infusion for active seizures or alcohol withdrawal;
○ Patients with evidence of active myocardial ischemia in prior 24 h;
○ Patients with evidence of increased intracranial pressure;
○ Decision to limit life-sustaining treatments.

**Figure 1:**
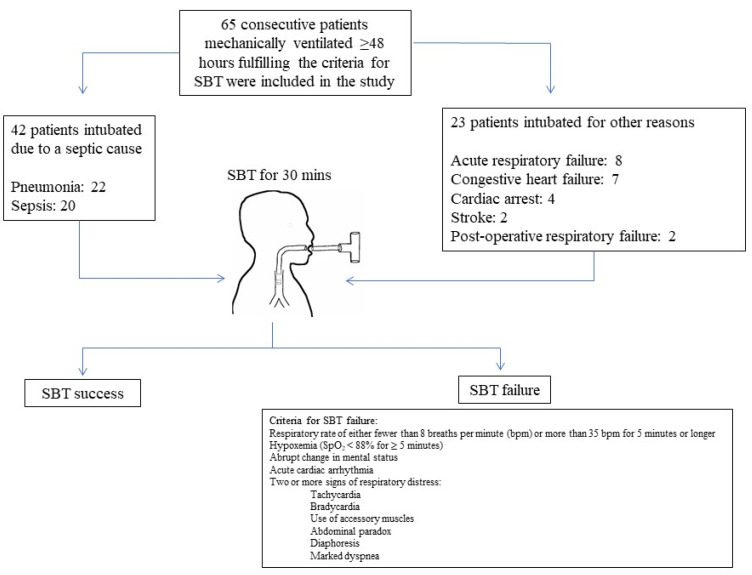
Consort diagram of patient screening.

Patients’ baseline characteristics are shown in Table 1. A consort diagram of patient screening is shown in Figure 1.

### 2.1. Study Protocol

Patients fulfilling the criteria for weaning were disconnected from ventilator and breathed spontaneously through a T-tube for a duration of 30 min. All patients received humidified oxygen with a FiO_2_ set at the same level as at ventilator. Concomitantly, they were connected in a pneumotach system to be recorded during the SBT. Respiratory rate, flow, and tidal volume during SBT were recorded, and rapid swallow breathing index (RSBI) was calculated, Table 1.

SBT failure was defined as:

i. Appearance of dyspnea or diaphoresis.
ii. Hypoxemia as defined with saturation of oxygen (SatO_2_) < 90% when FiO_2_ 0.4 and/or hypercapnia (PaCO_2_ ˃ 45 mmHg) or respiratory acidosis.
iii. Tachypnea (RR > 35/min) or tachycardia (HR > 140 beats/min) or increases or decreases in HR of more than 20% compared with mechanical ventilation.
iv. Hypertension or hypotension (increase or decrease of blood pressure >20% compared with mechanical ventilation.
v. Impaired consciousness or agitation.
vi. Use of auxiliary respiratory muscles or paradoxical thoraco-abdominal movement.

Blood samples were collected just before SBT (with the patient on mechanical ventilation), at the end of a 30 min SBT, and 24 h later. Serum was obtained after centrifuging the sample at 300× *g* (1800 rpm) for 15 min and it was stored at −80 °C. The determination of cytokine levels was performed with Enzyme-linked Immunosorbent Assay technique, (ELISA), following the manufacturer’s instructions (IL-18, IL-18BP, IL-6, and TNF: Invitrogen by Thermo Fischer Scientific, sensitivity 9 pg/mL, 20 pg/mL, and 0.92 pg/mL respectively; TNF-a: eBioscience, sensitivity 5.0 pg/mL).

### 2.2. mRNA Expression of IL-18

Blood collection. Venous blood (500 μL) was collected in the entrance and within the first 24 h post ICU admission. Samples were collected in RNA protect Animal Blood Tubes (Qiagen, Germany) and stored at −80 °C until used.

RNA isolation from blood. RNA extraction was performed using RNeasy Protect Animal Blood Kit (Qiagen), following the manufacturer’s instructions. Total RNA concentration and quality were determined spectrophotometrically at 260 and 280 nm, while RNA integrity was evaluated with formaldehyde agarose gel electrophoresis. Total RNA was stored at −80 °C until used.

RT-PCR. Total RNA (100 ng) from each sample were reverse-transcribed into single-stranded cDNA in a 10 μL reaction mixture, using Primescript RT reagent kit from Takara (Takara Bio Inc., Shiga, Japan), following the manufacturer’s instructions. The success of the synthesis of the single-stranded cDNA was tested by its PCR amplification. PCR was performed using 5.0 μL of cDNA, 1.5 mM MgCl_2_, 400 μM dNTPs, 500 nM primers, 1.5 U of Dream-Taq DNA polymerase and 1x reaction buffer (Fermentas, Thermofisher Scientific), in a PTC-200 thermocycler (MJ Research Inc., Waltham, MA, USA). Equal amounts (10 μL) of all amplicons were electrophoresed on a 2.5% agarose gel, visualized following RedGel staining (Biotium Inc., Hayward, CA, USA) and photographed under ultraviolet light with a Kodak DC120 digital camera (Kodak, Rochester, NY, USA).

Quantitative real-time PCR. A highly sensitive quantitative real-time PCR method has been developed for the quantification of both glyceraldehyde 3-phosphate dehydrogenase (GAPDH) and IL-18 mRNAs, with the use of SYBR^®^ Green Dye detection systems. For the amplification of GAPDH, as well as the IL-18 mRNA sequences, gene-specific sets of primers were designed according to the information on the National Centre for Biotechnology Information Sequence database and Primer Express program (Applied Biosystems, Thermofisher Scientific). GAPDH, 5’ ACATCGCTCAGACACCATGG-3’ (forward) and 5’GTAGTTGAGGTCAA-TGAAGGG-3’ (reverse), IL-18 5’-GCTTGAATCTAAA-TTATCAGTC-3’ (forward) and 5’-GAAGATTCAAATTGCATCTTAT-3’(reverse).

Quantitative real-time PCR analysis was performed in 96-well plates on a PTC-200 thermocycler (MJ Research Inc.). The 20 μL reaction mixture contained 10 ng cDNA, 300 nM primers and 2x KAPA SYBR^®^ Fast qPCR Master Mix Universal (Kapa Biosystems, Boston, MA, USA), in which a KAPA SYBR^®^ DNA polymerase is included. The thermal protocol conditions consisted of 2 min at 50 °C and 10 min at 95 °C polymerase activation step, 50 cycles of denaturation at 95 °C for 15 s, primer annealing at 54 °C for 30 s, extension at 72 °C for 30 s and a final extension step at 72 °C for 10 min. All samples were amplified in triplicate and the average CT values were calculated for their subsequent expression analysis. GAPDH expression was used for the normalization of *IL18* mRNA expression levels between samples from the different groups.

The study was approved by the “Sotiria” hospital Ethics Committee (Approval number: 37764). Patients’ data confidentiality was preserved in compliance with the Declaration of Helsinki.

### 2.3. Statistical Analysis

All data were analyzed using the statistical package for social science (SPSS) 10.0 for Windows program. All data were given as mean ± standard deviation (SD). Chi-square test was used to compare differences between the frequencies. Serum cytokines levels were analyzed using the normality test. The Mann–Whitney *U* and Student’s *t* test were used to compare mean values between groups. Spearman rank correlation test was used for the assessment of correlation. Statistical significance was accepted as *p* < 0.05.

Levels that were undetectable were assigned a value equal to the lower limit of detection for the assay.

## 3. Results

A successful SBT was achieved in 45 patients (69.2%). Septic patients had a higher percentage of SBT failure as compared to non-septic patients (85% vs. 15%, *p* = 0.026), with an odds ratio for failing 4.5 times that of non-septic patients (OR = 4.5 95%CI: 1.16–17.68, *p* 0.022). Clinical characteristics of patients according to SBT outcome are shown in Table 1 and Table 2. Fifty-one percent of the septic patients were admitted with pneumonia, while 47% of the patients were already septic upon admission. The most common causes of ICU admission in the non-septic group were acute or chronic respiratory failure, congestive heart failure, cardiac arrest, stroke, and post-operative respiratory failure.

Patients that failed as compared to those that succeeded SBT exhibited a higher respiratory rate (*p* < 0.001), higher heart rate (*p* < 0.053), higher PaCO2 (*p* < 0.003), lower pH (*p* < 0.003), and higher f/VT ratio (>105) (*p* = 0.021) at the end of the trial.

**Figure 2:**
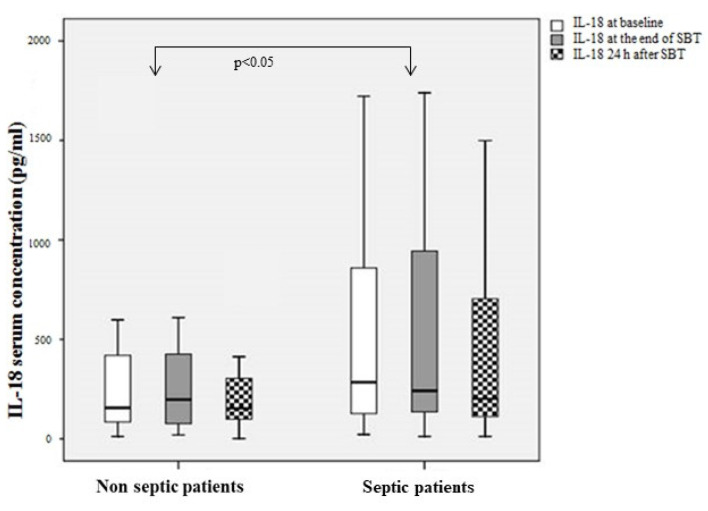
IL-18 serum levels at three time points—baseline, at 30 min SBT and at 24 h.

**Figure 3:**
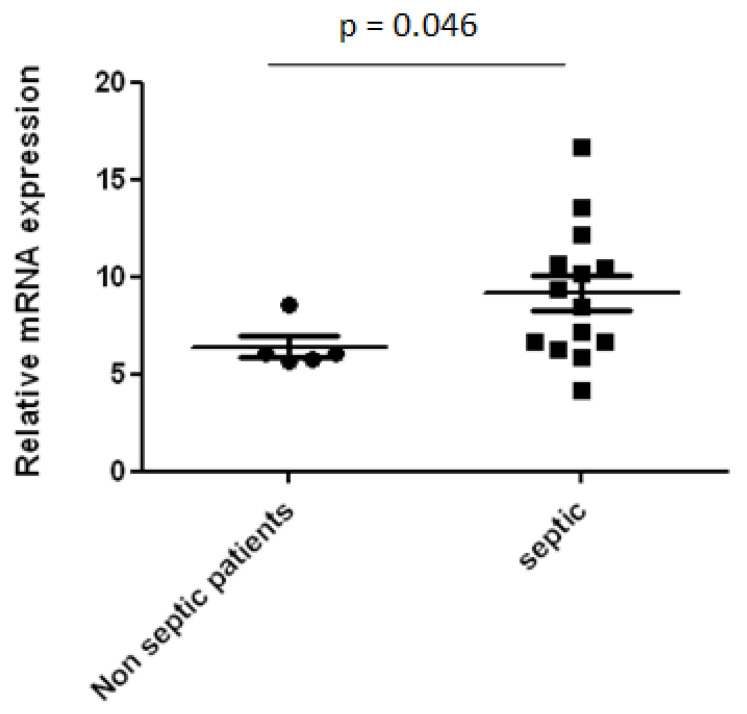
IL-18 mRNA expression in the serum of septic and non-septic patients.

**Figure 4:**
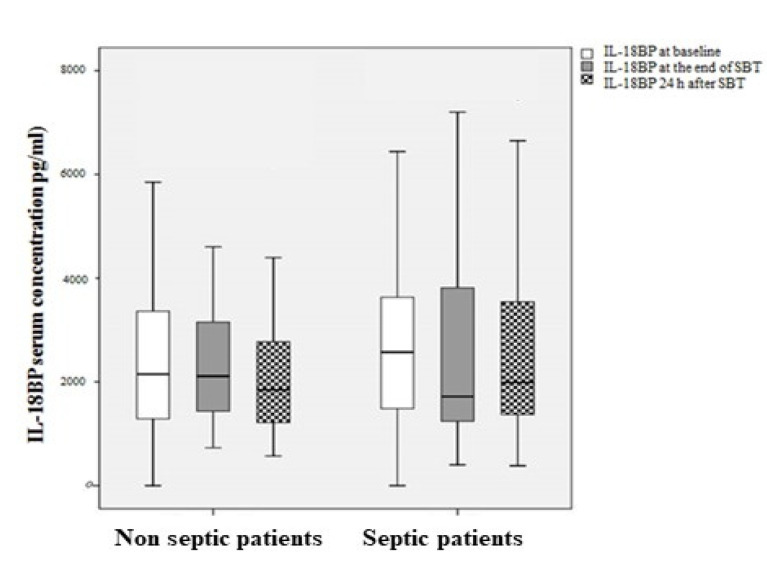
IL-18BP serum levels at three time points—baseline, at 30 min SBT and at 24 h.

IL-18 levels and the relative mRNA expression in serum were significantly higher in recovering septic as compared to non-septic patients at the time assessed by the clinicians as right for performing SBT (*p* < 0.05), Figure 2 and Figure 3. Serum IL-18BP levels were found to be almost 10-fold higher than IL-18 levels, Figure 4.

The kinetics of all of the pro-inflammatory cytokines assessed at the three time points according to outcome showed no significant differences. Sepsis was independently associated with higher serum IL-18 and TNF levels in two-time-point GEE models (before and at the end of SBT) adjusted for APACHE II score and age, with beta coefficients (95% CI, *p* values) as follows: IL-18 388 (53—723, *p* = 0.023), and TNF 32 (0.3—64, *p* = 0.048). IL-6 displayed a significant decrease in septic compared to non-septic patients in a two-time-point GEE model adjusted for age; beta coefficient 123 [95% CI −238 to −8, *p* = 0.036].

In a logistic regression model with SBT success as the dependent variable, RSBI (per unit increase) was independently associated with a 2% decrease in the probability of successful SBT (*p* = 0.011). A cut-off of 105 breaths/L/min for RSBI predicted a 5.6-fold higher probability of successful SBT, comparing patients below with patients above cut-off (95% CI: 1.4—22.2, *p* = 0.015). IL-18BP displayed an independent negative association with RSBI. IL-18BP serum levels displayed a mean decrease of 10.9 units per unit increase of RSBI (95% CI: −17.6 to −4, *p* = 0.002).

## 4. Discussion

Our study showed a 4.5-fold probability of failing SBT in patients recovering from sepsis as compared to non-septic patients. IL-18 levels and its relative mRNA expression in serum were found significantly higher in recovering septic as compared to their counter-parts non-septic patients. Sepsis was independently associated with higher serum levels of IL-18 and TNF at two time points (before and at the end of SBT). Furthermore, IL-18BP was independently negatively associated with rapid shallow breathing and SBT failure probability.

The relationship between systemic inflammation and SBT outcome seems to be partly related to the cardiopulmonary stress of patients during this process [28]. Sepsis, a condition which multiplies the inflammatory burden, is a known state that further affects the breathing pattern and weaning outcomes in critically ill patients [1,2]. Indeed, septic patients are dependent on mechanical ventilation for a longer time, and usually experience more weaning difficulties than patients without sepsis [2]. The adverse effect of sepsis was evident in our study as well, since recovering septic patients, despite fulfilling the criteria for SBT, had a significantly higher percentage of SBT failure as compared to non-septic patients (85% vs. 15%), and 4.5 times odds ratio of failing compared to their counterparts. Our findings coincide with other reported data showing that critically ill patients with sepsis are more likely to fail SBT and require more weaning days until successful extubation compared to non-septic critically ill patients [2,3,4,5,6]. These difficulties seem to be associated with the rapid and shallow breathing of these patients on SBT while recovering from sepsis and respiratory failure [3,4,5].

Sepsis results from an abnormal or dysregulated host response to infection via the activation of inflammasomes [29]. IL-18 is regarded as an important factor in the pathophysiology of sepsis with IL-18 levels found to be increased in septic patients in several studies [19,20,23,30]. Interestingly, we showed for the first time, that at the time that patients were considered as eligible for SBT, serum IL-18 levels and its relative mRNA expression increased in those recovering from sepsis and were significantly higher as compared to patients admitted for non-septic causes. It is evident that sustained sepsis inflammation occurs for a long time in the recovering septic patients and may interfere with the weaning difficulties in these patients. It is well documented that increased IL-18 levels are associated with worse severity and worse outcome of sepsis [19,20]. A direct correlation of IL-18 with APACHE II score which reflects severity [31] has been shown in previous studies. It is suggested that IL-18 could be used as a biomarker of monitoring sepsis severity throughout recovery in critically ill patients.

IL-18 and IL-18BP levels likely rise in parallel during pro-inflammatory states; however, there is still a relative excess of circulating free IL-18 in disease [27]. Indeed, serum IL-18BP levels were found to be almost 10-fold higher than IL-18 levels in our cohort. It is known that IL-18BP binds and neutralizes IL-18, therefore altering the levels of free IL-18, which is most likely the active moiety of interest [26]. Its role is protective, since it down-regulates the triggering of responses to routine infections and by binding to IL-18 lowers its levels and blunts its pro-inflammatory effects. In our study, IL-18BP displayed an independent negative association with RSBI probably reflecting its activity in binding to increasing levels of IL-18 during stress. This correlation underlines the relationship of these biomarkers with the stress of breathing. Several studies have associated RSBI during the SBT trial with SBT failure in patients recovering from sepsis and respiratory failure [2,3,4,5,6]. It is suggested that in these patients, RSBI occurs due to reduced respiratory pump capacity, which is less evident in non-septic patients [2]. Indeed, the negative impact of sepsis on respiratory muscle function has been demonstrated in several studies [32,33,34,35,36,37]. Interestingly, in our study, IL-18BP serum levels displayed a mean decrease of 10.9 units per unit increase of RSBI (95% CI: −17.6 to −4, *p* = 0.002). The strong correlation between IL-18BP (and not IL-1) and RSBI indicates IL-18BP as a potential biomarker of SBT failure. Unfortunately, due to the small sample size of septic patients in our study, a cut-off value of IL-18/IL-18BP levels indicating the recovery from sepsis, and possibly the opportunity of facing a successful SBT, could not be calculated.

The transition from mechanical ventilation to spontaneous breathing is a stressful procedure which results in an increase of respiratory muscle energy demands to cope with respiratory load [38]. The stress of failed SBT has been associated with an increase in systemic IL-6 in COPD patients [9], but this was not the case in non-COPD patients. We observed a significant decrease of IL-6 in septic compared to non-septic patients through-out failed SBT. We could suggest that an increase of IL-6 was not observed, possibly because our cohort of patients had a better reserve for coping with the SBT stress than COPD patients.

Although we did not observe a significant increase of cytokines at the end of SBT, we demonstrated that sepsis was independently associated with higher serum IL-18 and TNF levels at two time points (baseline, 30 min). In addition to IL-18, as discussed above, TNF also represents a key mediator of sepsis and sepsis-induced organ injury [39,40]. In the study of Shindoh et al., endotoxin induced TNF expression in the diaphragm after in vivo administration [41]. Up-regulation of pro-inflammatory cytokine messenger ribonucleic acid and protein levels in vivo and in vitro has also been reported for both diaphragm and limb muscles in response to LPS. Interestingly, higher levels were found in the diaphragm, suggesting that the respiratory muscles may be predisposed to pro-inflammatory responses [37] and that systemic cytokine response in sepsis also results in local amplification of pro-inflammatory cytokines in muscle. TNF may play an additional role in SBT failure, since it produces an immediate decrease in contractile muscle function, presumably due to alteration in contractile proteins [42]. Furthermore, it is well recognized that TNF causes muscle wasting in inflammatory processes [32].

We acknowledge that the main limitation of this study is the small sample size, which may have influenced the lack of statistically significant correlation of IL-18 with SBT failure, which was shown for IL-18BP. However, it is indirectly suggested. Secondly, another limitation could be the duration of SBT. It is possible that during a longer SBT (120 min), the kinetics of the cytokines might be more evident. A further increase might be demonstrated due to the progressive increase in stress in patients failing SBT.

In conclusion, our results underline the significance of the effect of a long-term sustained subclinical septic inflammatory state on weaning failure. We provide the first evidence of IL18/1IL-18BP implication in failed SBT in 30 min in patients recovering from sepsis. It is evident that we need biomarkers for the estimation of the septic burden to better estimate the right time for weaning. Further studies are needed to explore the potential predictive capacity of IL-18/IL-18BP of SBT outcome in septic patients.

## Figures and Tables

**Table 1:** Patients’ characteristics grouped by SBT outcome.

	Total (*n* = 65)	SBT Success (*n* = 45)	SBT Failure (*n* = 20)	*p* Value
Male sex (%)	36 (55.4%)	28 (62.2%)	8 (40%)	0.113
Age (yrs) (mean ± SD)	69.8 ± 13.3	69.8 ± 12	70 ± 16.2	0.962
APACHE II score (mean ± SD)	22.0 ± 7.5	21.6 ± 7.7	23.1 ± 6.9	0.474
Days in ICU until SBT (mean ± SD)	8.8 ± 6.3	8.8 ± 6.3	8.7 ± 5.6	0.956
Sepsis (%)	42 (64.6%)	25 (55.6%)	17 (85%)	0.026
WBC (k/mm^3^) (mean ± SD)	13,173 ± 5015	12,659 ± 4626	11,276 ± 3988	0.251
CRP (mg/dL) (mean ± SD)	10.5 ± 3.6	10.8 ± 8.8	11.7 ± 4.4	0.740
Albumin (g/dL) (mean ± SD)	2.6 ± 0.3	2.9 ± 0.4	2.7 ± 0.3	0.71
PCT (ng/mL) median (interquartile range)	0.38 (0.18–0.71)	0.37 (0.17–0.94)	0.40 (0.16–0.58)	0.611
VE MV (lt)	10.5 ± 1.9	10.4 ± 2.2	9.8 ± 1.2	0.373
VE (lt)	12.2 ± 4.7	12.2 ± 5.4	12.1 ± 3.2	0.982
f/VT (RSBI)	98.0 ± 60.8	80.0 ± 38.9	137.8 ± 80.9	0.021

SBT: spontaneous breathing trial; RSBI: rapid swallow breathing index.

**Table 2:** Changes in vital signs, lactate, and arterial blood gasses during SBT (*n* = 65).

		MV (PSV)	SBT	*p* Value *
RR	SBT failure	26 ± 5.7	32.4 ± 7.7	0.001
	SBT success	23.7 ± 5.6	24.5 ± 5.4	0.2
HR	SBT failure	90.2 ± 15.5	95.6 ± 14.1	0.053
	SBT success	88.2 ± 17.0	91.0 ± 15.9	<0.001
MAP	SBT failure	93.0 ± 14.7	96.8 ± 21.2	0.494
	SBT success	92.5 ± 15.6	88.5 ± 13.8	0.207
PaCO_2_	SBT failure	42.8 ± 6.8	49.1 ± 13.6	0.003
	SBT success	37.4 ± 6.9	37.2 ± 7.4	0.844
Arterial pH	SBT failure	7.46 ± 0.04	7.41± 0.07	0.003
	SBT success	7.45 ± 0.05	7.45 ± 0.04	0.774
PaO_2_	SBT failure	76.5 ± 12.1	83.9 ±34.2	0.794
	SBT success	84.1 ± 16.0	109.2 ± 33.9	<0.001
Lactate (mmol/L)	SBT failure	1.0 ± 0.3	0.9 ± 0.3	0.010
	SBT success	1.0 ± 0.4	1.0 ± 0.4	0.261

MV: mechanical ventilation; SBT: spontaneous breathing trial; MAP: mean arterial pressure; HR: heart rate; RR: respiratory rate; * Wilcoxon’s test.

## References

[1] Angus D.C., Linde-Zwirble W.T., Lidicker J., Clermont G., Carcillo J., Pinsky M.R. (2001). Epidemiology of severe sepsis in the United States: Analysis of incidence, outcome, and associated costs of care. Crit. Care Med..

[2] Amoateng-Adjepong Y., Jacob B.K., Ahmad M., Manthous C.A. (1997). The effect of sepsis on breathing pattern and weaning outcomes in patients recovering from respiratory failure. Chest.

[3] Yang K.L., Tobin M.J. (1991). A prospective study of indexes predicting the outcome of trials of weaning from mechanical ventilation. N. Engl. J. Med..

[4] Epstein S.K. (1995). Evaluation of the rapid shallow breathing index (RVR) in the clinical setting. Am. J. Respir. Crit. Care Med..

[5] Chatila W., Jacob B., Guaglionone D., Manthous C.A. (1996). The unassisted respiratory rate:tidal volume ratio accurately predicts weaning outcome. Am. J. Med..

[6] Eastwood P., Hillman D., Finucane K.E. (1994). Ventilator. Ventilatory responses to inspiratory threshold loading and role of muscle fatigue in task failure. J. Appl. Physiol..

[7] Thijs L.G., Hack C.E. (1995). Time course of cytokine levels in sepsis. Intensiv. Care Med..

[8] Claeys R., Vinken S., Spapen H., ver Elst K., Huyghens L., Gorus F.K. (2002). Plasma procalcitonin and C-reactive protein in acute septic shock: Clinical and biological correlates. Crit Care Med..

[9] Sellarés J., Loureiro H., Ferrer M., Amaro R., Farre R., Torres A. (2012). The effect of spontaneous breathing on systemic interleukin-6 during ventilator weaning. Eur. Respir. J..

[10] Forgiarini S.G.I., Rosa D., Forgiarini L.F., Teixeira C., Andrade C.F., Forgiarini L.A., Felix E.A., Friedman G. (2018). Evaluation of systemic inflammation in patients being weaned from mechanical ventilation. Clinics.

[11] Mikacenic C., Price B.L., Harju-Baker S., O’Mahony D.S., Robinson-Cohen C., Radella F., Hahn W.O., Katz R., Christiani D.C., Himmelfarb J. (2017). A Two-Biomarker Model Predicts Mortality in the Critically Ill with Sepsis. Am. J. Respir. Crit. Care Med..

[12] Barre M., Behnes M., Hamed S., Pauly D., Lepiorz D., Lang S., Akin I., Borggrefe M., Bertsch T., Hoffmann U. (2018). Revisiting the prognostic value of monocyte chemotactic protein 1 and interleukin-6 in the sepsis-3 era. J. Crit. Care.

[13] Yang C.-H., Hsiao J.-L., Wu M.-F., Lu M.-H., Chang H.-M., Ko W.-S., Chiou Y.-L. (2018). The declined levels of inflammatory cytokines related with weaning rate during period of septic patients using ventilators. Clin. Respir. J..

[14] Yamada T., Ishikawa M., Yamashita H., Fujiwara M., Usami M., Ueda T., Terashima M., Kohama K., Nakao A., Kotani J. (2014). IL18 production and IL18 promoter polymorphisms correlate with mortality in ICU patients. Vivo.

[15] Tabah A., Buetti N., Barbier F., Timsit J.F. (2021). Current opinion in management of septic shock due to Gram-negative bacteria. Curr. Opin. Infect. Dis..

[16] Gao Q., Li Z., Mo X., Wu Y., Zhou H., Peng J. (2021). Combined procalcitonin and hemogram parameters contribute to early differential diagnosis of Gram-negative/Gram-positive bloodstream infections. J. Clin. Lab. Anal..

[17] Shakoory B., Carcillo J.A., Chatham W.W., Amdur R.L., Zhao H., Dinarello C.A., Cron R.Q., Opal S.M. (2016). Interleukin-1 receptor blockade is associated with reduced mortality in sepsis patients with features of macrophage activation syndrome: Reanalysis of a prior phase III trial. Crit. Care Med..

[18] Dolinay T., Kim Y.S., Howrylak J., Hunninghake G.M., An C.H., Fredenburgh L., Massaro A.F., Rogers A., Gazourian L., Nakahira K. (2012). Inflammasome-regulated cytokines are critical mediators of acute lung injury. Am. J. Respir. Crit. Care Med..

[19] Rogers A.J., Guan J., Trtchounian A., Hunninghake G.M., Kaimal R., Desai M., Kozikowski L.A., DeSouza L., Mogan S., Liu K.D. (2019). Association of Elevated Plasma Interleukin-18 Level With Increased Mortality in a Clinical Trial of Statin Treatment for Acute Respiratory Distress Syndrome. Crit. Care Med..

[20] Mierzchala-Pasierb M., Krzystek-Korpacka M., Lesnik P., Adamik B., Placzkowska S., Serek P., Gamian A., Lipinska-Gediga M. (2019). Interleukin-18 serum levels in sepsis: Correlation with disease severity and inflammatory markers. Cytokine.

[21] Elsherbiny N.M., Al-Gayyar M.M.H. (2016). The role of IL-18 in type 1 diabetic nephropathy: The problem and future treatment. Cytokine.

[22] Ratsimandresy R.A., Indramohan M., Dorfleutner A., Stehlik C. (2017). The AIM2 inflammasome is a central regulator of intestinal homeostasis through the IL-18/IL-22/STAT3 pathway. Cell. Mol. Immunol..

[23] Wu Q., Xiao Z., Pu Y., Zhou J., Wang D., Huang Z., Hou D. (2019). TnI and IL-18 levels are associated with prognosis of sepsis. Postgrad. Med J..

[24] Wu H., Craft M.L., Wang P., Wyburn K.R., Chen G., Ma J., Hambly B., Chadban S.J. (2008). IL-18 contributes to renal damage after ischemia-reperfusion. J. Am. Soc. Nephrol. JASN.

[25] Kim S.-H., Eisenstein M., Reznikov L., Fantuzzi G., Novick D., Rubinstein M., Dinarello C.A. (2000). Structural requirements of six naturally occurring isoforms of the IL-18 binding protein to inhibit IL-18. Proc. Natl. Acad. Sci. USA.

[26] Dinarello C.A., Novick D., Kim S., Kaplanski G. (2013). Interleukin-18 and IL-18 binding protein. Front. Immunol..

[27] Novick D., Schwartsburd B., Pinkus R., Suissa D., Belzer I., Sthoeger Z., Keane W.F., Chvatchko Y., Kim S.H., Fantuzzi G. (2001). A novel IL-18BP ELISA shows elevated serum IL-18BP in sepsis and extensive decrease of free IL-18. Cytokine.

[28] de Oliveira R.P., Hetzel M.P., dos Anjos Silva M., Dallegrave D., Friedman G. (2010). Mechanical ventilation with high tidal volume induces inflammation in patients without lung disease. Crit. Care.

[29] Danielski L.G., Della Giustina A., Bonfante S., Barichello T., Petronilho F. (2020). The NLRP3 Inflammasome and Its Role in Sepsis Development. Inflammation.

[30] Chaudhry H., Zhou J., Zhong Y., Ali M.M., McGuire F., Nagarkatti P.S., Nagarkatti M. (2013). Role of cytokines as a double-edged sword in sepsis. Vivo.

[31] Godinjak A., Iglica A., Rama A., Tančica I., Jusufović S., Ajanović A., Kukuljac A. (2016). Predictive value of saps II and APACHE II scoring systems for patient outcome in medical intensive care unit. Acta Med. Acad..

[32] Callahan L.A., Supinski G.S. (2009). Sepsis-induced myopathy. Crit. Care Med..

[33] Rossignol B., Gueret G., Pennec J.-P., Morel J., Rannou F., Giroux-Metges M.-A., Talarmin H., Gioux M., Arvieux C.C. (2008). Effects of chronic sepsis on contractile properties of fast twitch muscle in an experimental model of critical illness neuromyopathy in the rat. Crit. Care Med..

[34] Hussain S.N., Simkus G., Roussos C. (1985). Respiratory muscle fatigue: A cause of ventilatory failure in septic shock. J. Appl. Physiol..

[35] Boczkowski J., Dureuil B., Branger C., Pavlovic D., Murciano D., Pariente R., Aubier M. (1988). Effects of sepsis on diaphragmatic function in rats. Am. Rev. Respir. Dis..

[36] Supinski G.S., Callahan L.A. (2006). Caspase activation contributes to endotoxin-induced diaphragm weakness. J. Appl. Physiol..

[37] Demoule A., Divangahi M., Yahiaoui L., Danialou G., Gvozdic D., Labbe K., Bao W., Petrof B.J. (2006). Endotoxin triggers nuclear factor-kappaB-dependent up-regulation of multiple proinflammatory genes in the diaphragm. Am. J. Respir. Crit. Care Med..

[38] Jubran A., Tobin M.J. (1997). Pathophysiologic basis of acute respiratory distress in patients who fail a trial of weaning from mechanical ventilation. Am. J. Respir. Crit. Care Med..

[39] Georgescu A.M., Banescu C., Azamfirei R., Hutanu A., Moldovan V., Badea I., Voidazan S., Dobreanu M., Chirtes I.R., Azamfirei L. (2020). Evaluation of TNF-alpha genetic polymorphisms as predictors for sepsis susceptibility and progression. BMC Infect. Dis..

[40] Roderburg C., Benz F., Schuller F., Pombeiro I., Hippe H.-J., Frey N., Trautwein C., Luedde T., Koch A., Tacke F. (2016). Serum Levels of TNF Receptor Ligands Are Dysregulated in Sepsis and Predict Mortality in Critically Ill Patients. PLoS ONE.

[41] Shindoh C., Hida W., Ohkawara Y., Yamauchi K., Ohno I., Takishima T., Shirato K. (1995). TNF-alpha mRNA expression in diaphragm muscle after endotoxin administration. Am. J. Respir. Crit. Care Med..

[42] Reid M.B., Lännergren J., Westerblad H. (2002). Respiratory and limb muscle weakness induced by tumor necrosis factor-alpha: Involvement of muscle myofilaments. Am. J. Respir. Crit. Care Med..

